# Controlled Design of a Robust Hierarchically Porous and Hollow Carbon Fiber Textile for High‐Performance Freestanding Electrodes

**DOI:** 10.1002/advs.201900762

**Published:** 2019-09-06

**Authors:** Quanxiang Li, Jiemin Wang, Chao Liu, Seyed Mousa Fakhrhoseini, Dan Liu, Liangzhu Zhang, Weiwei Lei, Minoo Naebe

**Affiliations:** ^1^ Institute for Frontier Materials Deakin University Waurn Ponds Campus, Locked Bag 20000 Geelong Victoria 3220 Australia

**Keywords:** freestanding electrodes, hierarchically porous carbon textiles, mechanically robust, ordered honeycomb‐like macropores

## Abstract

For most carbon‐based materials, hierarchical porous structure including well‐defined macropores, mesopores, and micropores is commonly seen in 3D aerogels, monoliths, or some carbothermic natural biomass. However, because of the filiform character and long draw ratio, it is difficult to achieve such pore network as well as attain excellent mechanical performance in a 1D single carbon fiber system. To address this issue, an innovative hierarchical porous and hollow carbon textile (HPHCT) is developed via the “dynamic template (KOH, SiO_2_, and Al_2_O_3_) calcination” strategy. Unlike conventional one‐step activated carbonized fiber simply with meso or micropores, the fabricated textile generates honeycomb‐like macropores uniformly spreading on fiber surface. More importantly, the ultra‐lightweight yet flexible HPHCT is mechanically robust, superior to ordinary carbonized one. In addition, it delivers high capacitance of maximum 220 F g^−1^ as well as keeping long term stability with 100% retention after 10 000 cycles as freestanding electrodes in supercapacitor. Meanwhile, the all‐solid integrated symmetric HPHCT supercapacitors demonstrates its high potential in powering electronics for wearable energy storage application.

## Graphical abstract

**The intensive explosion effect** is used to generate a hierarchically porous carbon fiber fabric with well‐defined pore dimensions and topologies. Such unique characteristics mean that the carbon fiber possesses high surface area yet robust mechanical properties, resulting in a flexible and efficient capacitive electrode.

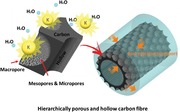

## 1 Introduction

Currently, hierarchically porous carbon species ranging from macroscopical carbonized monolith to microscopic carbon nanomaterials are attracting a great degree of interest.^1^ With unique structure‐dependent property, these carbon‐based structures offer unique opportunities in energy storage and electrocatalysis.^2^ As for electrode materials, hierarchical macro–meso–microporous structure presents more advantages compared to the carbon materials with simple or dual pore structures.^3^ This is mainly due to the minimized diffusive resistance of mass transport through macropores, as well as high surface area which lends itself effectively for dispersion of active sites over the micro and/or mesopores. Nevertheless, most hierarchical porous carbon materials are in the form of powders which needs the use of insulating binders, thereby limiting their applications in flexible and wearable electronics.^4^ To address this gap, a number of studies have focused on the design of porous carbon textile as the bind free electrodes.^5^ However, their poor mechanical performance hardly satisfy the requirement of wearable electronics, in particular, when a large number of macropores exist in one single carbon fiber.^6^ In addition, conventional pore forming approaches such as hard/soft templates, acid etching, air calcining, or CO_2_ activation usually result in micro‐ and mesopores in the fiber rather than interconnect ordered macroporous structure.[qv: 5a,c,d,g,7] As a result, it is very challenging for those methods to fully release the charge storage capability of carbon textile electrodes because they can only activate a very limited surface region (less than 50 nm deepness) of carbon fibers with tens of micrometer diameter. Moreover, the lack of interconnect ordered macroporous structure causes insufficient ionic diffusion resulting in unsatisfactory electrochemical performance.^8^ Therefore, fabrication of hierarchical porous yet mechanically robust textile remains a challenge.

Herein, using a novel method, we develop a hierarchical porous and hollow carbon textile (HPHCT) which exhibits high performance as a freestanding electrode for supercapacitors. Notably, in spite of highly porous features, the HPHCT is mechanically robust to bend, fold and twist. As a symmetric supercapacitor, both high volumetric power density 178 667 mW cm^−3^ and energy density 737 mWh cm^−3^ could be achieved. In addition, all‐solid integrated symmetric HPHCT supercapacitors also demonstrate a high potential in powering the portable electronics. The facile and innovative synthesis route, outstanding mechanical performance as well as the excellent capacitive performance make the developed textile structure promising for wearable energy storage applications.

## 2 Results and Discussion

### 2.1 Preparation of Highly Porous Hollow Textile

**Figure 1:**
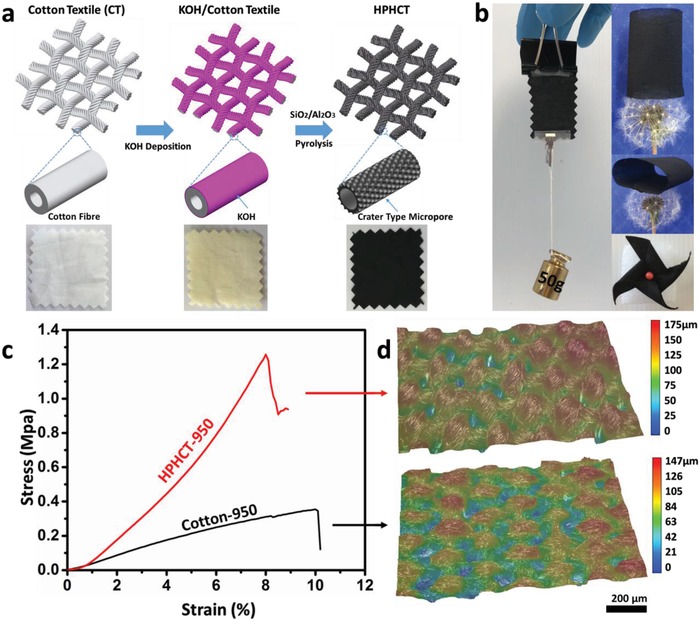
a) Schematic illustration demonstrating the preparation procedure of HPHCT with novel fiber surface structure from cotton textile. b) Digital photo of the lightweight, flexible, and strong HPHCT sitting on a dandelion and forming into a pinwheel c) Representative stress and strain curves of carbon textiles. d) 3D microscopy of carbon textiles.

**Figure** **1**a shows a schematic illustration of the novel process developed in this study to prepare 0.1 mm thick HPHCT from commercial 0.2 mm cotton fabric. The fabrication method is based on the carbonization of cotton fabric coated with KOH. Different from conventional KOH activation, both the Al_2_O_3_ loading panel and SiO_2_ tube affect the final architecture. The fabricated HPHCT is lightweight and flexible as well as being strong enough to be stretched, folded, and scrolled into the complex structures as demonstrated in Figure 1b.

Despite the highly hierarchical pore features, the prepared HPHCT possess a high tensile stress of ≈1.3 MPa and elastic modulus of ≈26.5 MPa, which is much higher than that of carbon textile obtained from pure cotton (Cotton‐950) (σ ≈ 0.35 MPa, *E* ≈ 4.9 MPa) (Figure 1c). From the microscope image (Figure 1d), it is found that the yarns in pristine cotton‐950 textile becomes loose after carbonization while the morphology of the HPHCT after the pyrolysis process remains intact with its original weave structures as well as interlacing warp and weft yarns in the vertical and horizontal directions. Although carbonization and activation process led to the serious cracking of individual carbon fibers, the highly rough surface can effectively prevent single fiber sliding and make yarn as well as the whole fabric even thicker and stronger than the carbon textile achieved from pure cotton.

### 2.2 Morphological, Microstructural, and Compositional Characteristics

**Figure 2:**
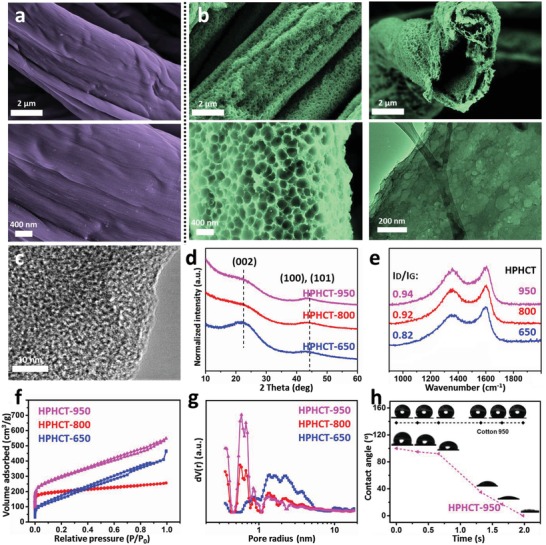
Surface morphology and phase structure characteristics of carbon textiles. a,b) SEM and TEM images of representative carbon textiles from a) Raw cotton textile, b) KOH coated cotton textile by novel porosity forming process at 950 °C, c) HRTEM images of HPHCT‐950, d,e) Phase structure identification from XRD and Raman spectra, f,g) Nitrogen adsorption/desorption isotherms and DFT pore distribution of textiles, and h) Dynamitic water contact angles of HPHCT‐950 and cotton‐950.

The morphologies and structures of the carbon fiber obtained from carbonizing cotton with and without the “dynamic template calcination” were examined by scanning electron microscope (SEM) and transmission electron microscopy (TEM). For pristine cotton, the surface of the resulted carbon fiber is relatively smooth and clean (**Figure** **2**a). For comparison, the carbon textile originating from conventional KOH activation process was characterized by SEM as well (Figure S1a, Supporting Information). It is found that the surface of carbon fiber activated by normal method is relatively smooth at low magnification, and some mesopores can be found at high magnification, which is very similar with the works reported by other researchers.[qv: 7a,9] In contrast, through our novel porosity forming process at 950 °C, many crater type macropores and mesopores were fabricated onto the carbon fiber surface (Figure 2b), and variation in surface morphology of fiber produced at different calcination temperature is shown in Figure S1b,c in the Supporting Information. Additionally, it has been shown that the highly porous carbon fiber has hollow structure, and the inner wall has less but similar hierarchical pores especially at the entrance to the cave. Generation of micro‐ and small mesoporosity on the fiber surface was further verified by high‐resolution transmission electron microscopy (HRTEM) (Figure 2c). The pores with pore radius less than 5 nm suggest the effectiveness of our dynamic template process in creating hierarchical macro–meso–microporous structure onto the fiber walls.

In X‐ray diffraction (XRD) spectra (Figure 2d), two broad and low‐intensity diffraction peaks at 2θ = 22.3° and 2θ = 43.8° are observed for the porous hollow textile, which can be indexed to (002) and (101) spacing of graphitic carbon (JCPDS No. 75‐1621).[qv: 7a,9] It is clear that the two peaks of HPHCT became broader with the increase of temperature, indicating a less ordered graphitic structure of HPHCT possibly due to excessive dynamic template activation. Figure 2e presents the Raman spectra of the carbon fiber within HPHCT, exhibiting defective D band (1355 cm^−1^) and graphitic G band (1600 cm^−1^) of carbon materials, respectively.^10^ The relative intensity ratio of D and G bands (*I*_D_/*I*_G_) suggests the degree of structural disorder with respect to a perfect graphitic structure. The change of *I*_D_/*I*_G_ with temperature‐dependent suggests that more caves and defect sites were introduced in HPHCT during the activation process, which is in good agreement with the XRD data.

**Figure 3:**
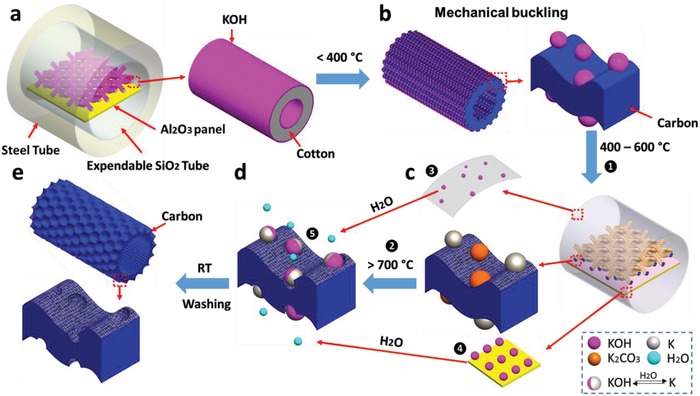
Proposed mechanism for the formation of hierarchical porous fiber surface. a) An expendable SiO_2_ tube containing KOH coated cotton textile lying on alumina panel was kept in stainless steel tube. b) The fiber experienced mechanical buckling and cellulose decomposition by pyrolysis at 250–400 °C. c) KOH activation process started on carbon fiber surface at 400–600 °C. d) The formation of crater type macropores by a series of reactions. e) A final porous hollow textile with novel fiber surface structure.

The Brunauer–Emmett–Teller (BET) surface area was further verified by N_2_ adsorption/desorption measurement (**Figure** **3**f). Among them, the HPHCT‐950 shows both the largest surface area (1120 m^2^ g^−1^) and high pore volume (0.49 cm^3^ g^−1^). While the pristine cotton textiles (cotton‐950) under 950 °C carbonization without activation only possess surface area of 438 m^2^ g^−1^ (Figure S2, Supporting Information). Remarkably, from density functional theory (DFT) pore distribution (Figure 3g), the dominating pores are small mesopores and micropores, which are more accessible to the ions in the electrolyte.[qv: 5f] In addition, higher pyrolysis temperatures lead to more defects and micropores, corresponding to the XRD and Raman analysis. Moreover, the highly porous and hollow structure also validates better water wettability. As shown in Figure 2h, the as‐obtained HPHCT‐950 exhibits good hydrophilicity whereas the cotton‐950 is hydrophobic. It is implied that the water can penetrate the macropore canal via capillary force,^11^ resulting in the deep wetting. As a result, the material is more accessible to the electrolyte when serving as electrode.[qv: 2a,12]

### 2.3 Mechanism Involved in Creation of Highly Porous and Hollow Textile

Historically, activated carbon and carbon textile with micro‐ and mesoporous morphologies have been produced by carbonizing cotton through different methods.^9^, ^13^ KOH is the most commonly used activating agent in the development of a porous structure. The activation process consumes carbon atoms and leaves behind abundant vacancies on the rest of carbon base. From the available literature, the surface morphologies of activated carbon textile are relatively smooth surface with plenty of pores with the size of 1–10 nanometers.[qv: 13b,14] In this work, we developed it by considerate design to create macrosize pores on the carbonized fiber as well as micro‐ and/or mesosize pores within those macropores. It is believed that this structure can provide more benefits for the application in supercapacitors, batteries, and electrocatalysis.

In order to understand the formation mechanism of the hierarchical macro–meso–microporous structure, a schematic of the proposed mechanism is illustrated in Figure 3. At the temperature less than 400 °C, cotton started to shrink due to α‐cellulose decomposition, being the building unit of cotton fibers. However, the shrinking rate of fiber surface is not uniform due to the presence of relatively rigid KOH layer as well as heterogeneity of nature material. As a result, the mechanical buckling occurs during pyrolysis process, and wrinkles can be found on the surface of carbonized textile, especially KOH coated textile. At the temperature above 400 °C, activation process commenced. The proposed chemical activation mechanism of KOH on carbon materials is as follows

(1) $$6\mathrm{KOH}+2C\to 2K+3H_{2}+2K_{2}{\mathrm{CO}}_{3}$$

According to literature, K_2_CO_3_ forms at ≈400 °C and KOH is completely consumed at ≈600 °C.^15^ With further increase in process temperature, i.e., >700 °C, the as‐formed K_2_CO_3_ can be decomposed into CO_2_ and K_2_O. The resulting CO_2_ can be further reduced by carbon to form CO at higher temperature. More importantly, the obtained K_2_O can be reduced by carbon to produce metallic potassium (K) at temperatures over 700 °C. Combining prominent global reaction, the reaction is proposed as shown in equation

(2) $$K_{2}{\mathrm{CO}}_{3}+2C\to 2K+3\mathrm{CO}$$

At the same time, KOH vapor was generated, spread out surrounding the sample in the quartz tube at high temperature (>400 °C). It is well‐known that KOH activation cannot be conducted in quartz tube furnace because KOH can react with silicon dioxide (SiO_2_) at high temperature according to the following equation

(3) $${\mathrm{SiO}}_{2}+2\mathrm{KOH}\to K_{2}{\mathrm{SiO}}_{3}+H_{2}O$$

Similarly, the alumina combustion panel used as the sample holder may also react with KOH at temperature above 700 °C based on the following equation

(4) $${\mathrm{Al}}_{2}O_{3}+2\mathrm{KOH}\to 2{\mathrm{KAlO}}_{2}+H_{2}O$$

However, it is important to note that light steam (H_2_O) is released from the reactions (3) and (4). Due to the extremely high hygroscopicity of K (formed as Equations (1) and (2)), the produced steam could be absorbed by metallic potassium particle. Consequently, on the surface of activated carbon, metallic potassium particles reacts rapidly and intensely with water and releases a significant amount of heat, as shown in Equation (5). The obtained KOH particle further activate carbon around repeatedly as Equations (1) and (2)

(5) $$\mathrm{2K}+2H_{2}O\to 2\mathrm{KOH}+H_{2}$$

As a result, the large honeycomb‐like macropores were obtained due to the continuous harsh explosion. Therefore, only KOH coated cotton samples together with Al_2_O_3_ panel and SiO_2_ tube are found to have hierarchical porous surface (Figure 2; Figure S1, Supporting Information). In order to better understand the formation mechanisms of hierarchical porous surface, more comparative experiments were conducted by using Al_2_O_3_ and SiO_2_ powder mixed with KOH coated cotton as well as KOH coated cotton textile covered by KOH pre‐etched quartz slide (Figure S1, Supporting Information). It can be seen that many honeycomb‐like macropores were fabricated onto the carbon fiber surface with different pore size by both methods. There were some particles embedded in the surface of carbon or larger pores at the area where cotton and quartz slide contacted each other, which further proved that the reaction between KOH and Al_2_O_3_ or SiO_2_ has effects on the formation of hierarchical porous fiber. Although our interpretation regarding the porous fiber formation mechanisms is simply based on the well‐known chemical reactions, it appears to correspond well with this relation.

### 2.4 Electrochemical Performances

**Figure 4:**
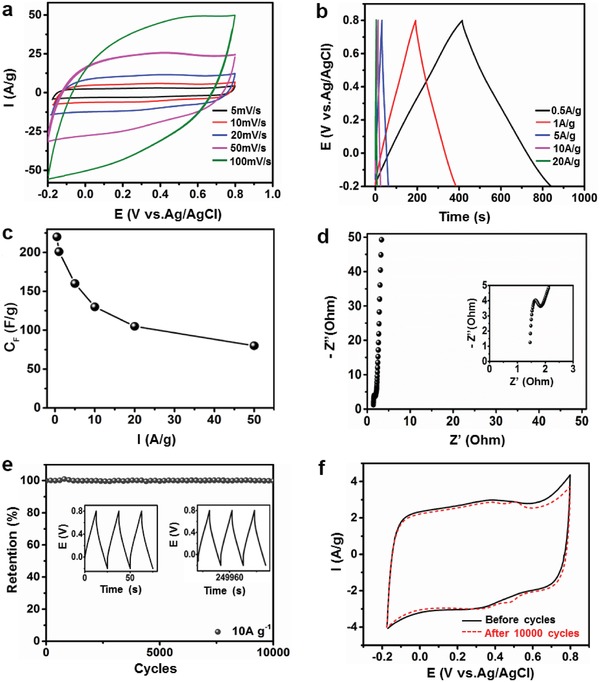
Electrochemical performances of a freestanding HPHCT‐950 electrode under three electrode system. a) CV curves at scan rate of 1–100 mV s^−1^. b) GCD curves at current density of 0.5–20 A g^−1^. c) Specific gravimetric capacitances at various current densities. d) Nyquist plots in the frequency ranging from 100 kHz to 10 mHz, the inset shows the semicircle shape of Nyquist ring towards higher frequency area. e) Capacitance retention during 10 000 cycles at 10 A g^−1^; inset shows GCD curves before and after cycling. f) CV curves before and after similar cycling obtained at a scan rate of 5 mV s^−1^.

First, the HPHCT‐950 is directly used as a freestanding electrode for supercapacitor in 1 m H_2_SO_4_ under a three‐electrode system. **Figure** **4**a exhibits the cyclic voltammetry (CV) curves at scan rates of 1, 5, 10, 20, 50, 100 mV s^−1^, respectively. It is clear that the quasi‐rectangular shapes are well kept, indicating the electrical double layer (EDL) capacitance of carbon materials.[qv: 4a] In addition, the shape remains rectangular even at higher scan rate, suggesting the fast kinetics for EDL formation and good stability of carbon materials.[qv: 3a] The galvanostatic charge–discharge (GCD) profiles are displayed in Figure 4b, the symmetrical GCD curves reflect a high degree of the reversibility for charge storage and delivery. More importantly, the freestanding HPHCT‐950 electrode delivers a high gravimetric capacitance (*C*_F_) of 220 F g^−1^ at a current density of 0.5 A g^−1^, which is greater than most reported carbon textile based materials (Table S3, Supporting Information). Furthermore, most freestanding carbon‐based electrodes would be deactivated under larger current charging (above 10 A g^−1^), due to the collapse of structure and poor wettability. However, for HPHCT‐950, the *C*_F_ of 80 F g^−1^ is maintained even at a large current density of 50 A g^−1^, which is promising for flexible carbon‐based freestanding electrodes. We also calculate the areal capacitances (*C*_S_) and volumetric capacitance (*C*_V_) according to the area and thickness of the textile, respectively (Figure S5, Supporting Information). It is highly encouraging that the HPHCT‐950 shows both high areal and volumetric capacitances at high current capacitances (Figure 4c; Figure S5, Supporting Information). This is due to the lightweight and high surface area of the architecture as well as the thin in depth of the textile. In fact, the hollow structure and honeycomb‐like macropores leads to better electrolyte wettability. Meanwhile, the micropores facilitates the ion and electron transport. As a result, more charges are accessible throughout the electrodes, thereby enhancing the capacitance largely. This is further demonstrated by electrochemical impedance spectroscopy (EIS) (Figure 4d). The almost inclined vertical line in low frequency region of Nyquist plots reveals the fast ion diffusion and migration in the HPHCT‐950. While the semicircle with a small diameter in high frequency area reflects the low internal charge‐transfer resistance during faradic reactions.[qv: 3b,4a] Besides, the electrode also performs great cycle stability (Figure 4e). Conventionally, some carbonized freestanding electrodes would crumble and collapse after long‐term use. However, despite highly porous and hollow feature, the HPHCT‐950 is still mechanically robust, which contributes to the good durability and antifatigue property. It can be seen that a 100% retention is achieved after 10 000 cycles. The nearly unchanged CV shapes (Figure 4f) and Nyquist plots (Figure S6, Supporting Information) before and after 10 000 cycles further demonstrates the great stability of HPHCT‐950 electrodes, which is prominent for energy storage.

**Figure 5:**
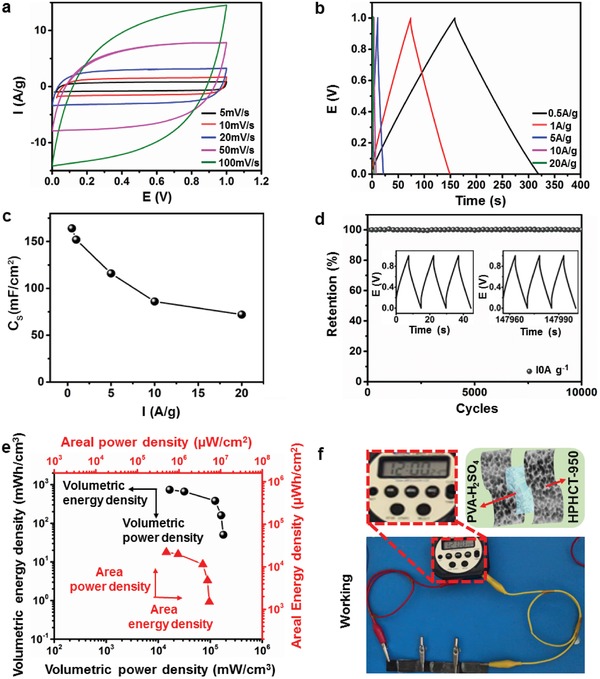
Electrochemical performances of HPHCT‐950 electrodes under symmetric electrode system. a) CV curves at scan rates of 5–100 mV s^−1^. b) GCD curves at current densities of 0.5–20 A g^−1^. c) Areal specific capacitances at various current densities. d) Capacitance retention during 10 000 cycles at 10 A g^−1^; inset shows GCD curves before and after cycling. e) Ragone plots (areal and volumetric) of HPHCT‐950. f) Photographs of a calculator powered using three tandem all‐solid sandwich‐structured symmetrical HPHCT‐950 supercapacitors.

The capacitive performance of a symmetrical HPHCT‐950 based supercapacitor is tested in a two‐electrode configuration in 1 m H_2_SO_4_. Likewise, the CV curves from 5 to 100 mV s^−1^ also show rectangular shapes, highlighting the EDL mechanism (**Figure** **5**a). The GCD plots exhibit generally symmetric, suggesting a high degree of reversibility (Figure 5b). Although the calculated gravimetric capacitance is 82 F g^−1^ at 0.5 A g^−1^ due to the cell configuration (Figure S7, Supporting Information). The areal capacitance of 164 mF cm^−2^ is still larger than reported textiles (Figure 5c) (Table S3, Supporting Information). Additionally, the symmetric electrodes supercapacitor also shows long cycle durability with 100% retention after 10 000 cycles (Figure 5d). And the CV curves and Nyquist plots nearly remain the same, confirming the great stability again (Figures S6 and S8, Supporting Information). According to the areal and volumetric capacitances, the corresponding energy density and power density are calculated in Figure 5e, respectively. The maximum volumetric power density could reach 178 667 mW cm^−3^ at energy density of 50 mWh cm^−3^. And the maximum energy density is 737 mWh cm^−3^ with power density of 16 600 mW cm^−3^. Similarly, for the areal power density and energy density, a maximum power density of 5.36 × 10^6^ µW cm^−2^ (energy density: 1510 µWh cm^−2^) and energy density of 22 100 µWh cm^−2^ (power density: 498 000 µW cm^−2^) could be achieved respectively. The freestanding electrodes deliver high volumetric/areal energy and power densities, in spite of the moderate gravimetric one (Figure S9, Supporting Information). These results originate from several aspects. First, the material is ultralight with hierarchical porous hollow structure, which enables more space to contact with electrolyte in a relatively low mass. Furthermore, the freestanding cloth electrode is thin enough (0.15 mm), improving the efficiency for spatial charging/discharging. In addition, the HPHCT‐950 constitutes of pure carbon materials rather than any metallic oxides such as MnO_2_ and NiCo_2_O_4_,^16^ which facilities the high surface area and low weight. Therefore, it exhibits great volumetric/area power and energy density. In this regards, we can use the freestanding HPHCT‐950 electrodes to fabricate all‐solid‐supercapacitors. In most studies, pure carbon‐based all‐solid‐supercapacitor is difficult to power electronics due to the poor energy and power densities and often pseudocapacitance materials or heteroatoms doping are used for improvement. However, this usually negatively affects the long‐term sustainability and increase the cost. Here, we simply use three tandem freestanding HPHCT‐950 to successfully power a calculator (Figure 5f). Notably, in this process, no binding agents such as PVDF, soft substrates such as cellulose and PET or metal current collector such as Ti mesh are used. This all carbon electrode based supercapacitor is soft, portable and environmentally friendly, satisfying the need for mass production of energy storage devices in industry.

## 3 Conclusion

In conclusion, we designed a novel “dynamic activation” route to successfully achieve robust hierarchically porous and hollow textiles with interconnected honeycomb‐like macropores. Through a series of experiments, the mechanism was well investigated, which interprets that the KOH, SiO_2_, and Al_2_O_3_ involved in calcination process plays an important role in creating hierarchical macro–meso–microporous fiber surface with significantly improved surface area and pore volume. Although the produced HPHCT possess highly porous and hollow architecture, it demonstrates a great deal of flexibility and mechanical robustness. More importantly, the pure carbon‐based material with high capacitance could serve as an efficient freestanding electrode for supercapacitors. The lightweight, high‐flexibility, and long‐term cyclicity in electrolyte make this material an excellent and promising candidate for energy storage application.

## 4 Experimental Section

*Fabrication of Carbon Textiles*: All of the chemicals were obtained from Sigma‐Aldrich (Sydney, Australia) and used “as received.” The commercially available natural cotton textile (cotton 100%) was first cleaned using distilled water in an ultrasonic bath before activation. Initially, the cotton textile was cut into pieces of 5 × 5 cm^2^. To prepare KOH coated cotton the cotton textile was mixed into 2% KOH aqueous solution under 60 °C. This mixture was stirred very gently for 12 h to ensure a uniform coating of KOH onto cotton fiber surface. The wet textile was then placed on a flat alumina panel and dried in an air‐circulating oven at 80 °C for 5 h. Before the carbonization process into a steel tube, the KOH‐treated cotton textile together with the alumina panel were first inserted into an expendable quartz tube for 1 h in argon atmosphere with a heating rate of 10 °C min^−1^. After cooling down to room temperature, the as‐obtained activated carbonized cotton were washed with 1% HCl (hydrochloric acid)/water solution and distilled water, respectively ,and then dried at 80 °C for 5 h. For comparison, activated carbon textile was prepared through the traditional method. Basically the prepared KOH‐treated cotton textile was carbonized at 950 °C in steel tube with no quartz tube.

*Characterization*: The morphologies of the samples were examined by a scanning electron microscope (ZEISS Supra 55 SEM VP) operated with a 5 kV accelerating voltage and 7.5 mm working distance. Samples were not coated prior to imaging. The microstructures of the samples were observed on a JEOL JEM2100 LaB6 (JEOL Ltd., Akishima, Japan) operated at an acceleration voltage of 200 kV. Powder X‐ray diffraction (with the 2θ from 10 to 60°) was measured to study the crystallinity of the samples. The surface of representative carbon textiles was analyzed with Renishaw inVia Raman microscope. Three random spots on each sample were picked to calculate change in D/G ratios of samples. Nitrogen adsorption and desorption isotherms were collected with a Tristar 3000 apparatus at 77 K. The mechanical property of carbon textile samples was determined using a Favimat fiber tester (Textechno H. Stein, Germany), and a minimum of five measurements were conducted for each sample. The contact angles are measured by using contact angle goniometer (CAM101, KSV).

*Electrochemical Measurements*: The electrochemical measurements were carried out in a three‐electrode system with Pt wire as the counter electrode, Ag/AgCl as the reference electrode in 1 m H_2_SO_4_ aqueous solution at room temperature. The freestanding HPHCT was used as working electrode directly. In the article, CV and GCD tests were conducted using Garmy 600 electrochemical workstation between −0.2 and 0.8 V (vs Ag/AgCl) at scan rates from 1 to100 mV s^−1^ and the current densities of 0.5–50 A g^−1^, respectively. The gravimetric, areal and volumetric capacitances of the active materials were calculated from constant charge/discharge curves by following equations

(6) $$C_{F}\;=\;\frac{I_{\mathrm{cons}}t}{m\Delta V}$$

(7) $$C_{S}\;=\;\frac{I_{\mathrm{cons}}t}{S\Delta V}$$

(8) $$C_{V}\;=\;\frac{I_{\mathrm{cons}}t}{V\Delta V}$$

where *C*_F_, *C*_S_, and *C*_V_ are the gravimetric capacitance (F g^−1^), areal capacitance (mF cm^−2^), and volumetric capacitance (F cm^−3^) respectively. *I*_cons_ is the constant discharge current (A). *t* is the discharge time (s), *m* is the active mass in the electrode (g), *S* is the active mass in the electrode (cm^2^), and *V* is the volume of the electrode (cm^3^). Δ*V* is the voltage range.

The cycling stability was studied at 10 A g^−1^ within a voltage window of −0.2–0.8 V (vs Ag/AgCl).

EIS was measured in the range of 10 mHz and 100 kHz with peak to peak amplitude of 10 mV at an open‐circuit potential.

For two‐electrode tests, two pieces of freestanding HPHCT with identical sample mass loading were assembled in 1 m H_2_SO_4_. CV and GCD tests were conducted using Garmy 600 electrochemical workstation between 0 and 1 V at scan rates from 1 to100 mV s^−1^ and the current densities of 0.5–20 A g^−1^, respectively. The gravimetric, areal and volumetric density and power density for the Ragone plots were calculated via the following equations, respectively

Gravimetric:

(9) $$E_{F}\;=\;\frac{C_{F}V^{2}}{2\;\times 3.6}$$

(10) $$P_{F}\;=\;\frac{E_{F}}{t}\times 3600$$

Areal:

(11) $$E_{S}\;=\;\frac{C_{S}V^{2}}{2\;\times 3.6}$$

(12) $$P_{S}\;=\;\frac{E_{S}}{t}\times 3600$$

Volumetric:

(13) $$E_{V}\;=\;\frac{C_{V}V^{2}}{2\;\times 3.6}$$

(14) $$P_{V}\;=\;\frac{E_{V}}{t}\times 3600$$

where *V* is the voltage after subtracting the ohmic drop (V) and *t* is the discharge time (h).

For the fabrication of the all‐solid sandwich‐structured symmetrical HPHCT950 supercapacitor, two pieces of HPHCT950 cloth were sandwiched with PVA and H_2_SO_4_ solid electrolyte. Then the supercapacitor was sealed and packed with Scotch tapes.

## Conflict of Interest

The authors declare no conflict of interest.

## Supporting information

Supplementary
