# Supplementary material for: Controlled Design of a Robust Hierarchically Porous and Hollow Carbon Fiber Textile for High‐Performance Freestanding Electrodes
Source: Adv Sci (Weinh). 2019 Sep 6;6(21):1900762. doi: 10.1002/advs.201900762 (PMC6839622; doi:10.1002/advs.201900762)
Supplement: Supplementary file 1 — Supplementary [file ADVS-6-1900762-s001.pdf]

Copyright WILEY-VCH Verlag GmbH & Co. KGaA, 69469 Weinheim, Germany, 2019.

ADVANCED SCIENCE Open Access

# Supporting Information

for *Adv. Sci.,* DOI: 10.1002/advs.201900762

# Controlled Design of a Robust Hierarchically Porous and Hollow Carbon Fiber Textile for High-Performance Freestanding Electrodes

*Quanxiang Li, Jiemin Wang, Chao Liu, Seyed Mousa Fakhrhoseini, Dan Liu, Liangzhu Zhang, Weiwei Lei,* and Minoo Naebe**

# Supporting information

**Controlled Design of a Robust Hierarchically Porous and Hollow Carbon Fiber Textile for High-Performance Freestanding Electrodes**

*Quanxiang Li,* †[a] *Jiemin Wang,* †[a] *Chao Liu,* [a] *Seyed Mousa Fakhrhoseini,* [a] *Dan Liu,* [a] *Liangzhu Zhang,* [a] *Weiwei Lei* [a,]* *and Minoo Naebe* [a,]*

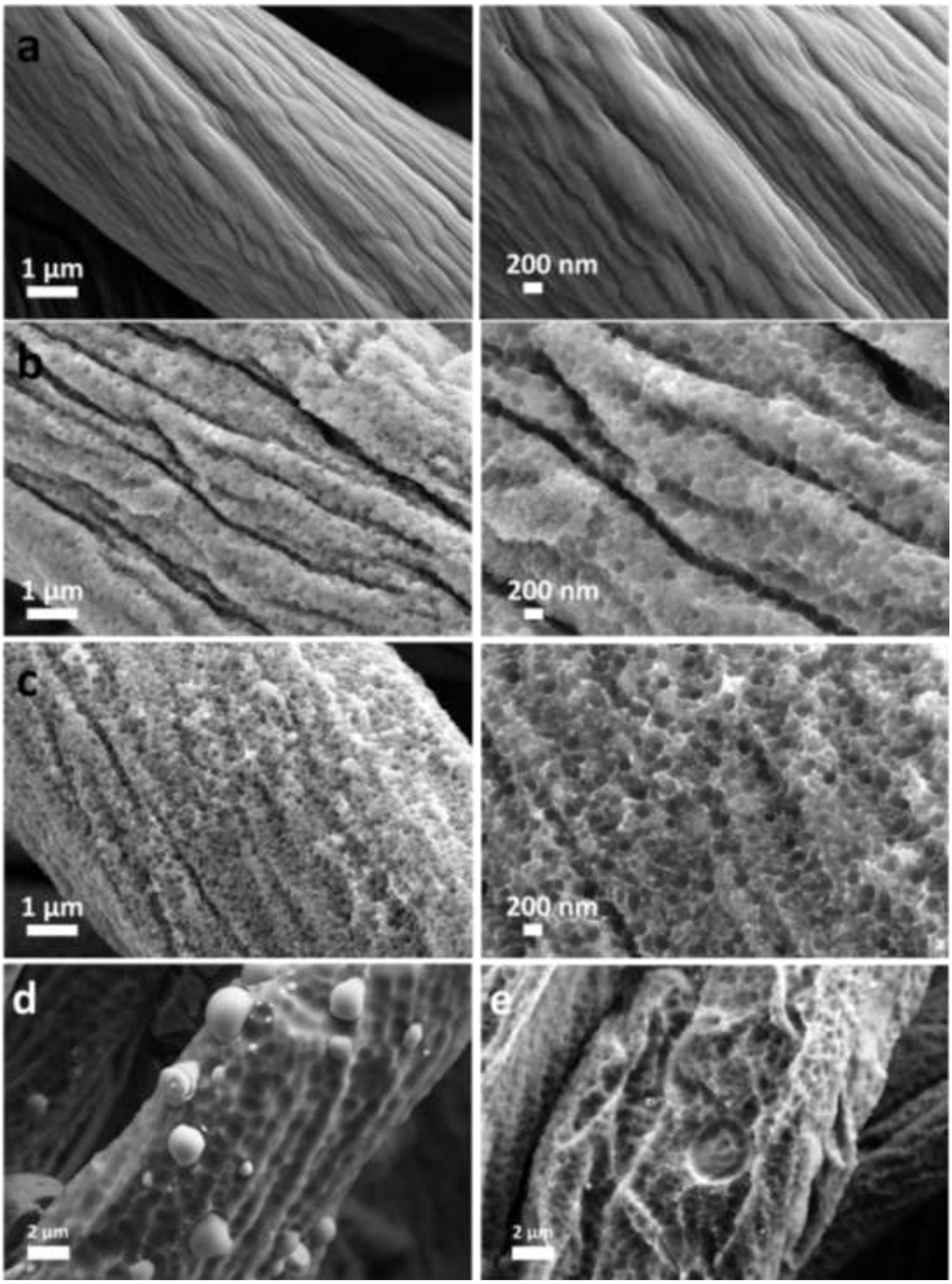


**Figure S1** Surface morphology of the representative carbon textiles from a) KOH coated cotton fabric by traditional method at 950 °C, b and c) KOH coated cotton textile by novel activation process at 650 °C and 800 °C, d) $Al_2O_3$ and $SiO_2$ powder mixed with KOH coated cotton textile and e) KOH coated cotton textile covered by quartz slide.

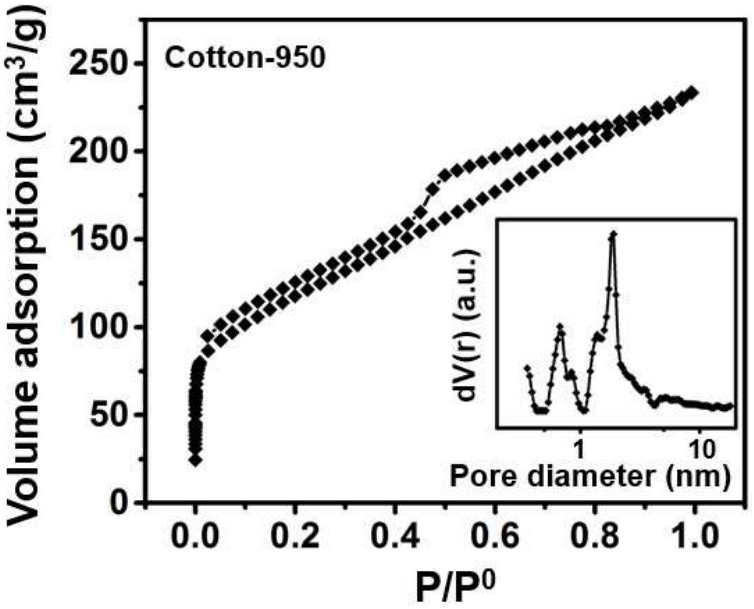


**Figure S2.** Nitrogen adsorption/desorption isotherms and pore distribution of cotton-950.

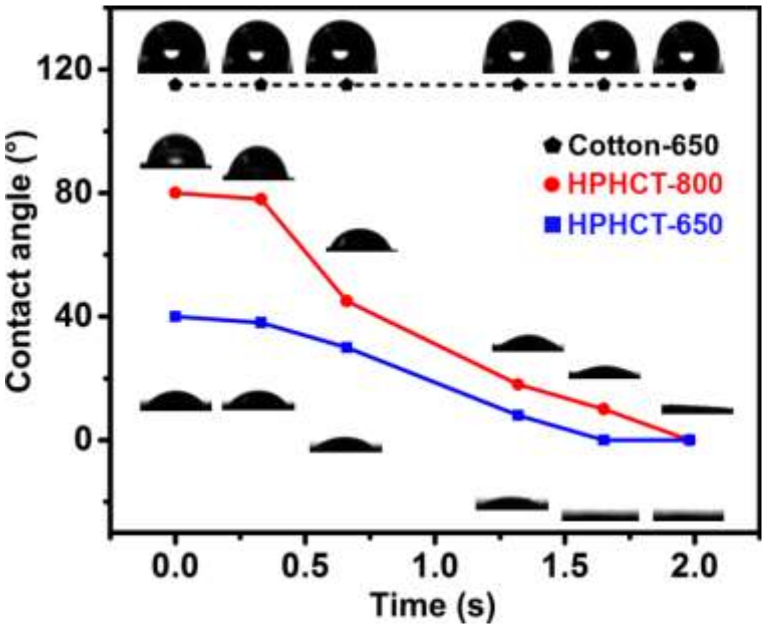


**Figure S3.** Dynamic Water contact angles of cotton-650, HPHCT-650 and HPHCT-800.

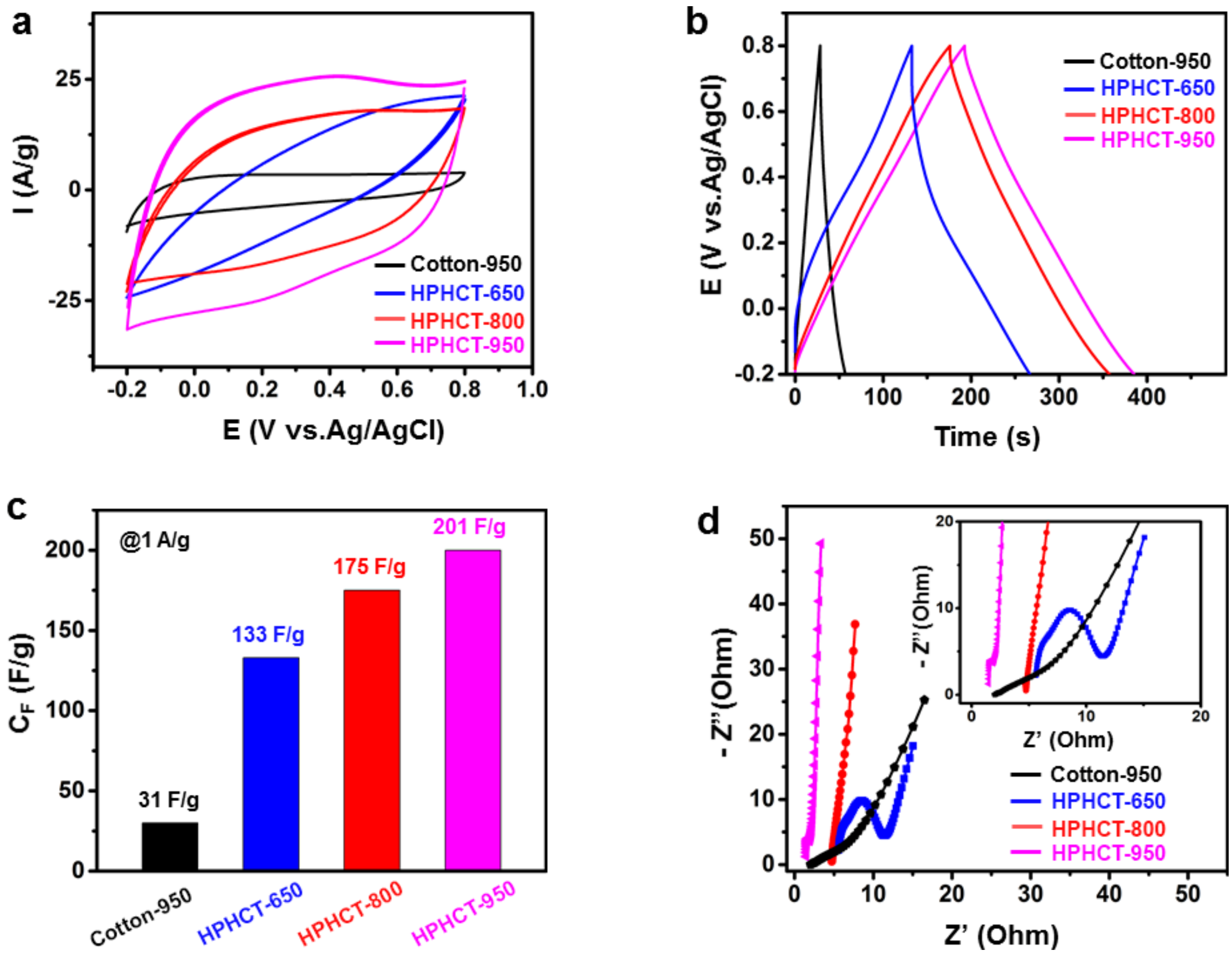


**Figure S4.** Electrodes performances of cotton-950, HPHCT-650, HPHCT-800 and HPHCT-950 for supercapacitors. a) CV curves at 50 mV/s, b) GCD curves at 1 A/g, c) Capacitance comparison, d) Nyquist plots comparison.

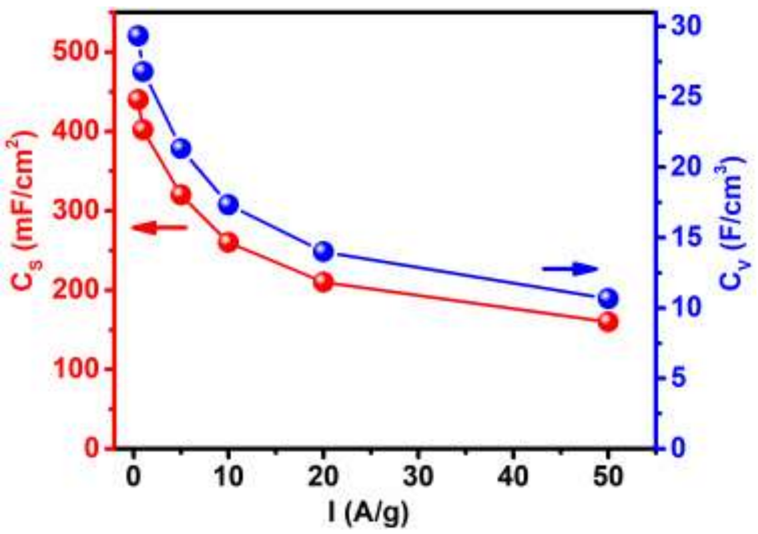


**Figure S5.** Specific capacitances (areal and volumetric) at various current densities in three electrodes system.

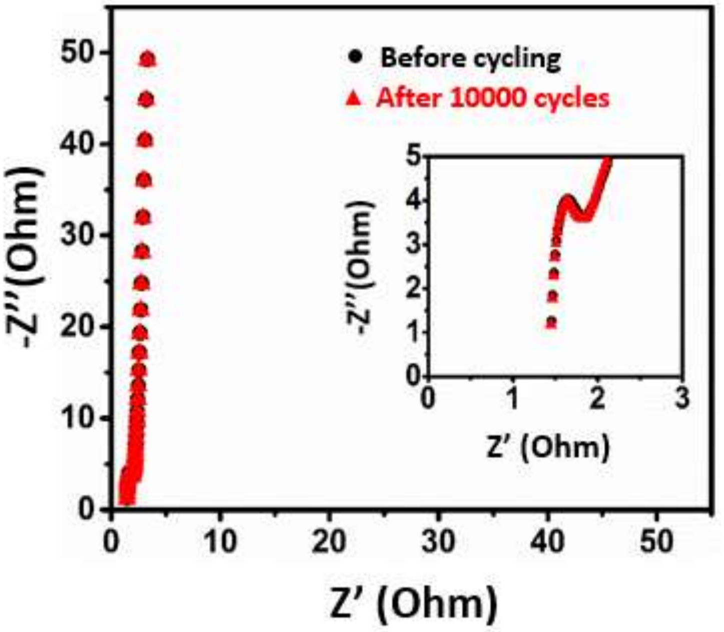


**Figure S6**. Nyquist plots before and after 10000 cycles in the 3-electrode system in the frequency ranging from 100 kHz to 10 mHz, the inset shows the semi-circle shape of Nyquist ring towards higher frequency area.

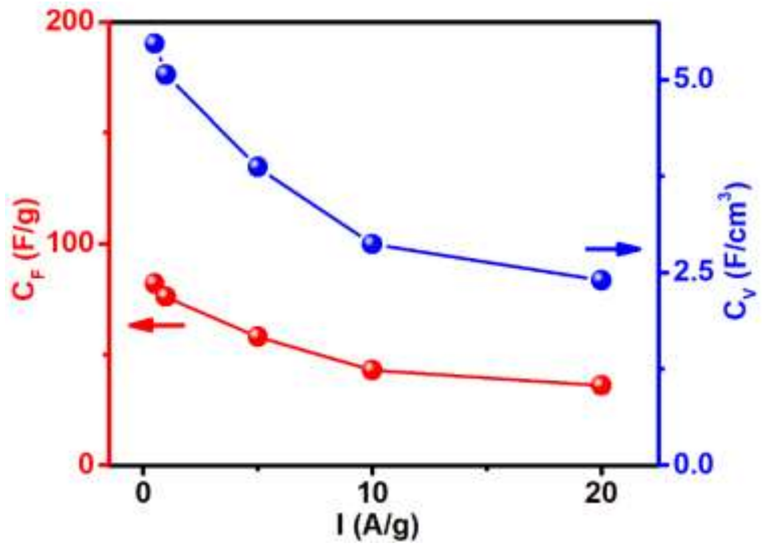


Figure S7. Specific capacitances (gravimetric and volumetric) at various current densities in two electrodes system.

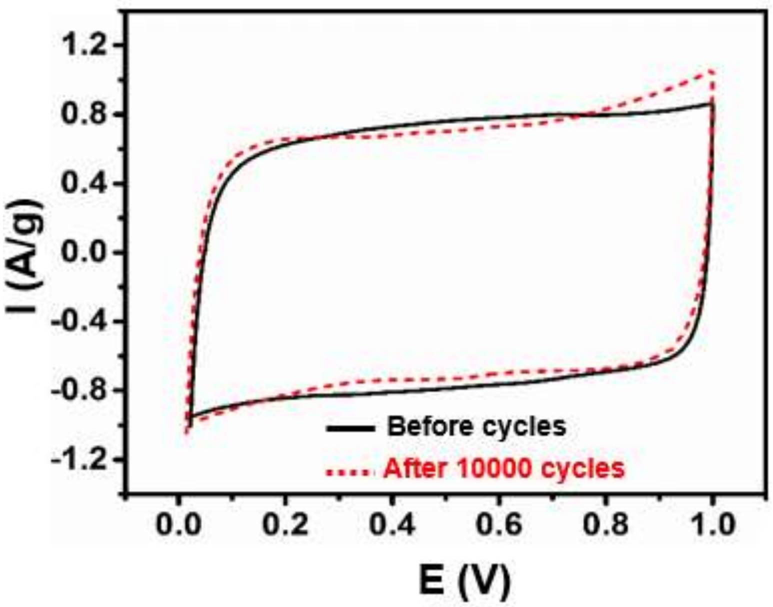


**Figure S8.** CV curves before and after 10000 cycles in the 2-electrode symmetric system.

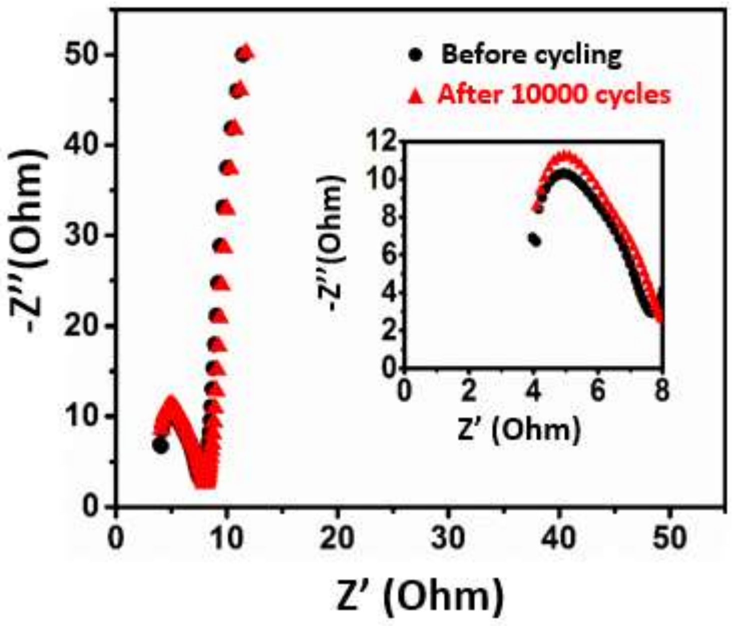


**Figure S9.** Nyquist plots before and after 10000 cycles in the 2-electrode symmetric system in the frequency ranging from 100 kHz to 10 mHz, the inset shows the semi-circle shape of Nyquist ring towards higher frequency area.

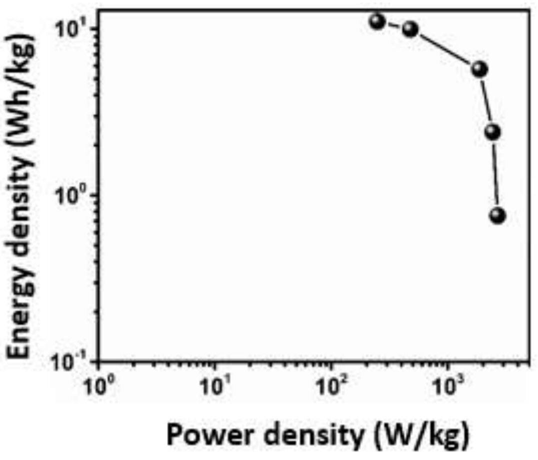


**Figure S10.** Gravimetric Ragone plots of HPHCT-950

**Table S1. The surface areas and pore volumes of textiles in this work.**

| Samples | Surface area ($m^2/g$) | Pore volume ($cm^3/g$) |
|---|---|---|
| **Cotton 950** | 438 | 0.329 |
| **HPHCT-650** | 613 | 0.364 |
| **HPHCT-800** | 745 | 0.412 |
| **HPHCT-950** | 1120 | 0.491 |

**Table S2. The electrical resistivity of textiles in this work.**

| Samples | Electrical resistivity (Ω/cm) |
|---|---|
| **Cotton 950** | 50 |
| **HPHCT-650** | 460 |
| **HPHCT-800** | 40 |
| **HPHCT-950** | 16 |

**Table S3 Capacitive performance comparison of HPHCT950 and other carbon fiber or cotton derived materials.**

| Samples | Systems | $C_F$ (F/g) | $C_S$ (mF/cm$^2$) | $C_V$ (F/cm$^3$) | Freestanding | Ref |
|---|---|---|---|---|---|---|
| **HPHCT-950** | 3-electrode | 220 | 440 | 29.3 | Yes | This work |
| **HPHCT-950** | 2-electrode | 82 | 164 | 5.5 | Yes | This work |
| **NOC-3** | 3-electrode | 180 | 290 | | No | 15 |
| **P-CNFs** | 3-electrode | 104 | | | Yes | 17 |
| **NCC-1h** | 3-electrode | 207 | | | Yes | 18 |
| **CNF/GN-2** | 3-electrode | 215 | | | Yes | 19 |
| **CNF/GN-2** | 2-electrode | 178 | 43.5 | 0.1451 | Yes | 19 |
| **CSHPC4** | 3-electrode | 175 | | | No | 21 |
| **p-a1000CT** | 2-electrode | 63.2 | 121.5 | | Yes | 22 |
| **ZFO-ACFs** | 3-electrode | 195 | | | No | 26 |
| **ZFO-ACFs** | 2-electrode | 45 | | | No | 26 |
